# 6D-QSAR for predicting biological activity of human aldose reductase inhibitors using quasar receptor surface modeling

**DOI:** 10.1186/s13065-023-00970-x

**Published:** 2023-06-22

**Authors:** Babak Sokouti, Maryam Hamzeh-Mivehroud

**Affiliations:** 1 Biotechnology Research Center, Tabriz University of Medical Sciences, Tabriz, Iran; 2 School of Pharmacy, Tabriz University of Medical Sciences, Tabriz, Iran

**Keywords:** Biological activity, Molecular descriptors, Multidimensional QSAR, Human aldose reductase inhibitors, 6D-QSAR

## Abstract

The application of QSAR analysis dates back a half-century ago and is currently continuously employed in any rational drug design. The multi-dimensional QSAR modeling can be a promising tool for researchers to develop reliable predictive QSAR models for designing novel compounds. In the present work, we studied inhibitors of human aldose reductase (AR) to generate multi-dimensional QSAR models using 3D- and 6D-QSAR methods. For this purpose, Pentacle and Quasar’s programs were used to produce the QSAR models using corresponding dissociation constant (K_d_) values. By inspecting the performance metrics of the generated models, we achieved similar results with comparable internal validation statistics. However, considering the externally validated values, 6D-QSAR models provide significantly better prediction of endpoint values. The obtained results suggest that the higher the dimension of the QSAR model, the higher the performance of the generated model. However, more studies are required to verify these outcomes.

**Supplementary Information:**

The online version contains supplementary material available at 10.1186/s13065-023-00970-x.

## Introduction

Targeted drug design is an inseparable part of any pharmaceutical industry, primarily achievable through various computer-aided drug design (CADD) approaches. Computational approaches are time-saving and cost-effective, which are two essential parameters in the drug development pipeline. The CADD approaches are divided into structure-based and ligand-based methods. Spatial analyses of interactions between ligand and target molecules is a core hypothesis of structure-based drug design. This approach’s main goal is to optimize the binding pattern of the studied ligand and corresponding receptor in a three-dimensional fashion. Therefore, having structural information on receptors can be helpful in structure-based computer-aided drug design investigations. In the case of the ligand-based approach, the ligands’ most important physicochemical, electronic, and conformational features are selected as significant contributors to biological activities. However, in this strategy, knowledge of the target structure is not needed for generating predictive models [1–3]. Tirofiban [4], zanamivir [5], boceprevir [6], saquinavir [7], captopril [8, 9], and aliskiren [10] are approved examples of marketed drugs using CADD strategies.

Quantitative structure-activity relationship (QSAR) is a valuable technique in CADD aiming to relate the generated predictive mathematical models using structural features and biological activity values. From a dimensional point of view, the type of QSAR models depends on the employed descriptors ranging from 0D-QSAR to 7D-QSAR. Several descriptors (e.g., atomic properties, fragment counts, and topological descriptors) constitute the components of 0D to 2D-QSAR. A 3D-QSAR analysis results from incorporating three-dimensional based descriptors considering an extra dimension in spatial coordinates. Additional dimensions to 3D-QSAR models necessitate the involvement of multivariate molecular descriptors based on conformational flexibility, induced fit, solvation function, and target-based receptor models. Such incorporations lead to the generation of multi-dimensional QSAR (i.e., 4D- to 7D-QSAR) [11]. Today, increasing interest is seen in using classic and 3D-QSAR models against the small number of studies employing multi-dimensional QSAR approaches along with their predictive power. However, it is not certain whether the higher the dimensionality, the higher the performance. In this respect, there is controversy in the technical literature about the performance of QSAR in terms of dimensionality. Therefore, more cases are needed to assess this debatable issue. Hence, we aimed to develop predictive multi-dimensional QSAR models using inhibitors of aldose reductase (AR) (EC 1.1.1.21) and evaluate their performance in the predictive capability of biological activities. AR is an oxidoreductase enzyme classified as the Aldo-Keto reductases (AKRs) superfamily responsible for the metabolism of glucose to sorbitol via the polyol pathway. However, in hyperglycemia, overproduction of sorbitol results in diabetic complications such as neuropathy, retinopathy, nephropathy, and cataractogenesis. Therefore, inhibition of AR could prevent such pathological events. In addition, AR inhibitors can be useful in inflammatory complications [12–16]. Accordingly, the prediction of the biological activity of AR inhibitors could pave the way for designing and developing novel compounds where the inhibition of AR is of paramount importance. Besides, by applying different multi-dimensional QSAR tools in predicting biological activity, it is possible to investigate the effect of 6D-QSAR as a representative of the multi-dimensional QSAR model compared to 3D-QSAR.

## Results

In this study, we employed two different multi-dimensional QSAR approaches (i.e., 3D-QSAR and 6D-QSAR) to predict the biological activity of aldose reductase inhibitors and evaluate the effect of additional dimensions on the predictive power of the generated models. In the case of 3D-QSAR, the bioactive conformations of the studied compounds were achieved by the 3D-solved structure of the co-crystallized ligand in complex with aldose reductase enzyme. The training and test sets were randomly selected based on their inhibitory activities, where both sets covered a similar range of activities. A 3D-QSAR model was trained using GRIND-based descriptors by applying the PLS model. Few rounds of the fractional factorial design (FFD) method were applied to improve the statistical quality of the obtained model until no observation of significant changes occurred in terms of squared correlation coefficient (R^2^), cross-validated correlation coefficient (q^2^), and standard deviation of the error of prediction (SDEP). Based on the obtained results, three latent variables (3LVs) were chosen as an optimum number of the PLS model. Table 1 gives the statistics for the 3D-QSAR model based on PLS analysis for AR inhibitors. Moreover, cross-validation methods, including LOO and LTO, are reproducible after several runs. However, in the case of RG, several runs with different seeds were conducted. Eventually, the average values of R^2^ and Q^2^ were 0.98 and 0.85, respectively, which are not significantly different from those mentioned in Table 1 (R^2^ = 0.98 and Q^2^ = 0.87).

**Table 1:** Statistics for the 3D-QSAR model based on PLS analysis for AR inhibitors

LV	SSX	SSX_acc_	SDEC	SDEP	R^2^	${\mathrm R}^{2}_{\mathrm{acc}}$	(LOO)	${\mathrm{Q}}^{2}_{\mathrm{acc}}$ (LTO)	${\mathrm{Q}}^{2}_{\mathrm{acc}}$ (5RG)
1	63.31	63.31	0.69	0.89	0.55	0.55	0.26	0.22	0.25
2	14.37	77.68	0.21	0.43	0.41	0.96	0.83	0.75	0.81
3	10.60	88.28	0.16	0.36	0.02	0.98	0.88	0.81	0.87

**Fig. 1:**
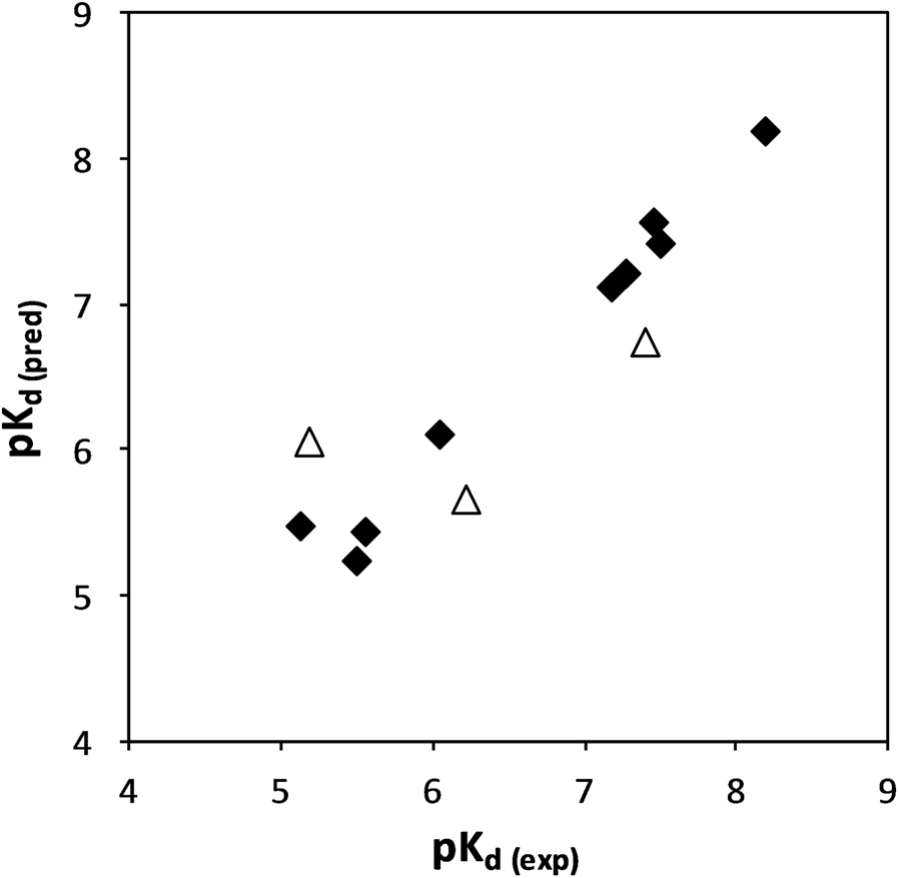
Experimental vs. predicted pK_d_ for the human aldose reductase inhibitors using 3D-QSAR based model. Filled diamonds indicate training set and open triangles show the test set compounds

Experimental vs. predicted values obtained from the 3D-QSAR analysis are given in Table 2 and illustrated in Fig. 1.

**Table 2:** Observed dissociation constant, pK_d(exp)_ of the human aldose reductase (AR) inhibitors vs. their predicted values using alignment independent 3D-QSAR analysis

Compound	pK_d(exp)_	pK_d(pred)_
a1	8.18	8.19
a2	7.17	7.12
a3	6.04	6.11
a4	7.45	7.56
a5^a^	5.18	6.06
a6	5.55	5.45
a7	5.12	5.49
a8	7.27	7.21
a9^a^	6.21	5.67
a10	5.49	5.25
a11	7.49	7.42
a12^a^	7.40	6.74
R^2^	0.98	
Q^2^_(LOO)_	0.88	
R^2^_Test_	0.42	

*SSX* X variable explanation, *SDEC* standard deviation of error of calculation, *SDEP* standard deviation of error of prediction

The acc stands for accumulative value, Validation methods used for calculation of q_2_ are: leave one out (LOO), leave two out (LTO) and random groups (RG)

^a^Test set

**Fig. 2:**
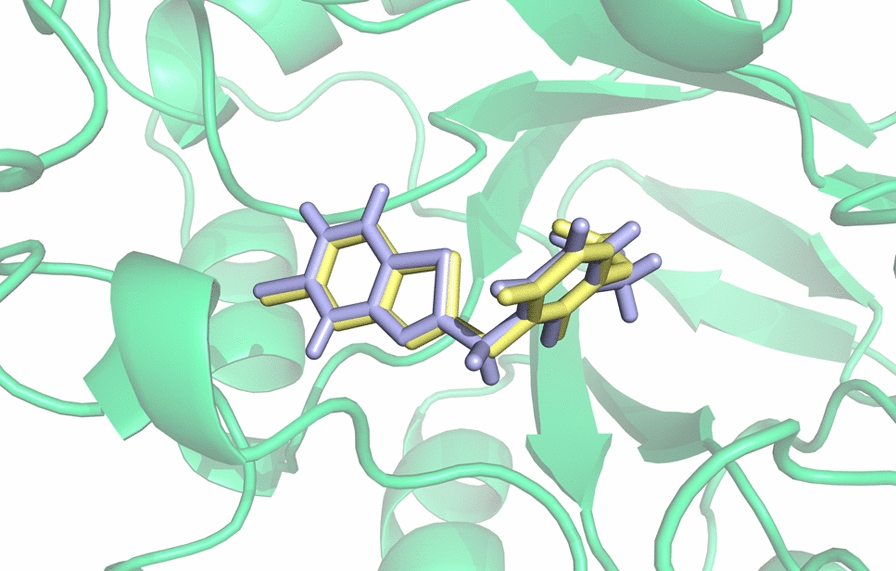
The docked and co-crystallized ligand (a4, PDB ID: 2PDG) using GOLD program (version 5.2.2) depicted by PyMOL software (version 1.1r1)

**Fig. 3:**
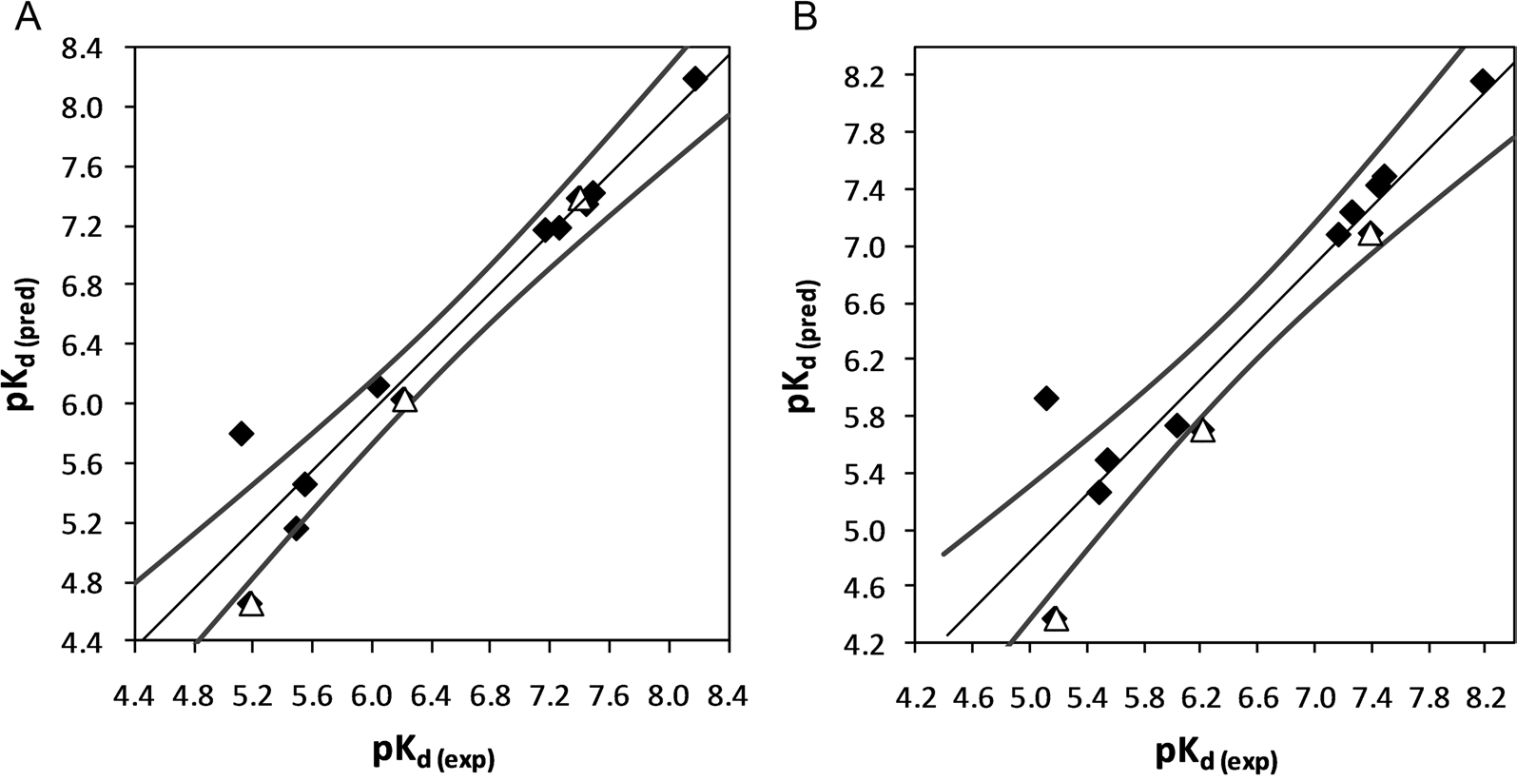
Experimental vs. predicted pK_d_ for the human aldose reductase inhibitors using 6D-QSAR based models. **A** and **B** are related to the results obtained from GOLD and AutoDock, respectively. Filled diamonds and open triangles are training and test sets, respectively

Molecular docking was conducted using two software, i.e., GOLD and AutoDock, which were used for generating an ensemble of different conformations of the studied ligands to be used in the Quasar program for developing the 6D-QSAR model. As a representative of molecular structures, the best solution for the docked pose of a4 compound (PDB ID: 2PDG) is depicted in Fig. 2. The calculated RMSD between docked and co-crystallized ligand is 0.289 Å. Table 3 shows the statistical results of the generated 6D-QSAR models using both conformation ensembles docked by the two mentioned algorithms. Figure 3 represents the scatter plot of experimental vs. predicted pK_d_ values, and the corresponding values are given in Table 3. It is worth mentioning that the prediction values (including all the statistical parameters, R^2^, Q^2^_LOO_, and R^2^_test_ in Table 3) are average values of 100 individual runs attained from the Quasar software.

**Table 3:** Observed dissociation constant, pK_d(exp)_ of the human aldose reductase (AR) inhibitors vs. their predicted values using GOLD and AutoDock through 6D-QSAR analysis

Compound	pK_d(exp)_	pK_d(pred_GOLD)_	pK_d(pred_AutoDock)_
a1	8.18	8.18	8.16
a2	7.17	7.16	7.08
a3	6.04	6.12	5.73
a4	7.45	7.34	7.43
a5^a^	5.18	4.65	4.37
a6	5.55	5.45	5.49
a7	5.12	5.79	5.92
a8	7.27	7.18	7.24
a9^a^	6.21	6.02	5.70
a10	5.49	5.16	5.26
a11	7.49	7.41	7.49
a12^a^	7.40	7.38	7.09
R^2^	–	0.92	0.92
Q^2^_(LOO)_	–	0.90	0.89
R^2^_Test_	–	0.76	0.58
RMS deviation training set	–	0.35	0.40
Maximal deviation training set	–	0.90	1.07
RMS deviation test set	–	0.44	0.78
Maximal deviation test set	–	0.71	1.09

Statistical parameters for the performance of predictive obtained models are also listed

*LOO* leave-one-out

^a^Test set

The results of analyses obtained from 3D- and 6D-QSAR models are presented in Tables 2 and 3. As can be seen, although the coefficient of determination (R^2^) for these models are comparable that are greater than 0.9 (i.e., 0.98 for 3D-QSAR and 0.92 for 6D-QSAR models) with marginally similar internal validations (3D-QSAR-Q^2^_LOO_: 0.88, 6D-QSAR_GOLD_-Q^2^_LOO_: 0.90, and 6D-QSAR_AutoDock_ -Q^2^_LOO_: 0.89), the external validations are significantly different in terms of binding affinity prediction of test dataset (3D-QSAR-R^2^_Test_: 0.42, 6D-QSAR_GOLD_-R^2^_Test_: 0.76, and 6D-QSAR_AutoDock_-R^2^_Test_: 0.58). Based on the obtained results, increasing the dimensionality in QSAR models may help improve prediction performance in the developed models. More details on the results are listed in Additional file 1.

**Fig. 4:**
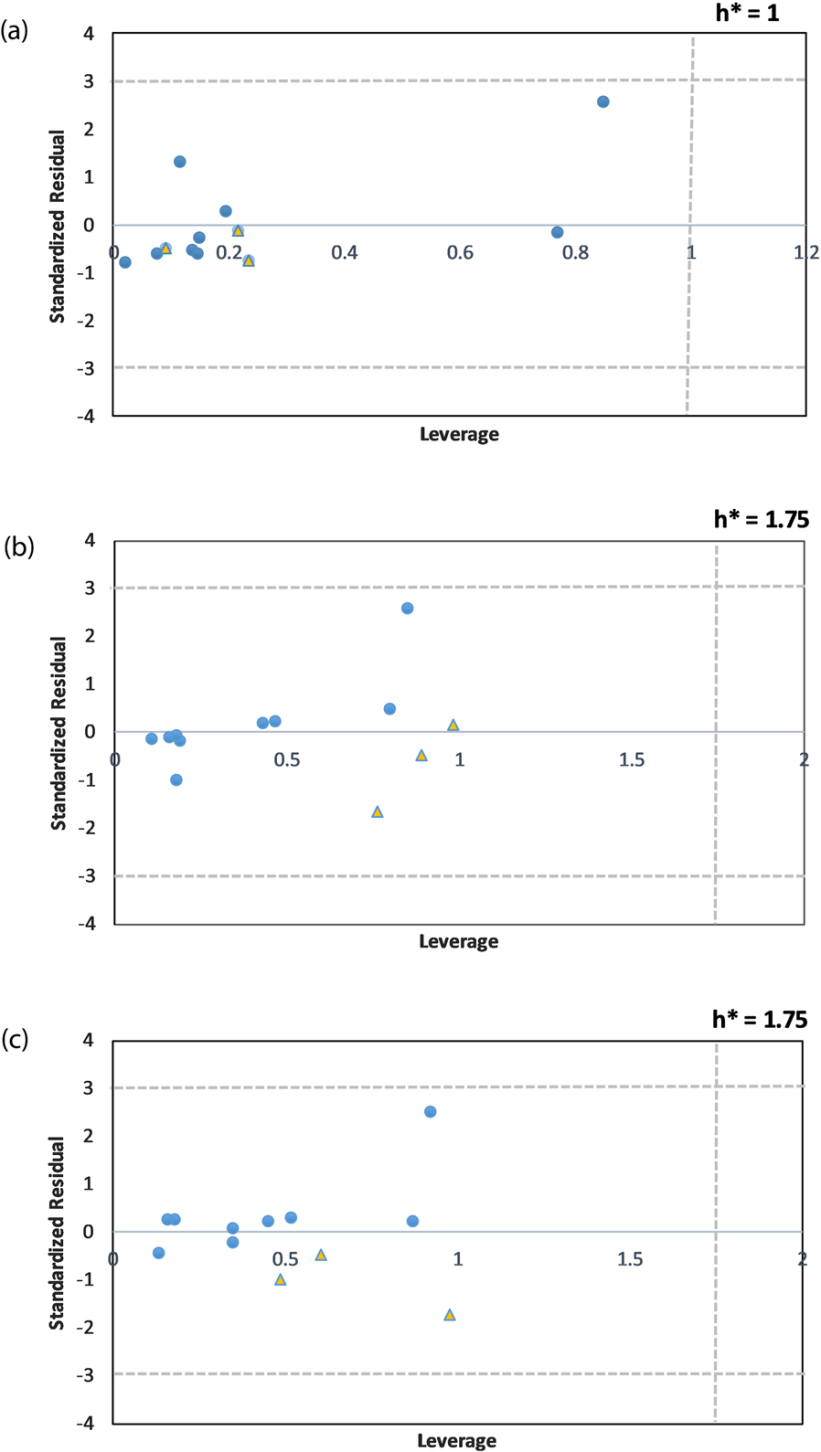
Applicability domain using Williams plot for (**a**) 3D-QSAR model, (**b**) 6D-QSAR model (GOLD), and (**c**) 6D-QSAR model (AutoDock). Standardized residuals are plotted vs. the leverage values for each compound. Circle and triangle shapes are used to demonstrate training and test dataset

The applicability domain (AD) was also determined for the studied molecules to detect the outlier in the defined chemical space. The Williams plots for the three developed QSAR models are demonstrated in Fig. 4. The illustrations suggest the absence of outliers, and all data points are within the AD range for all generated QSAR models.

## Discussion

Bridging from bench to bedside for a new chemical entity necessitates employing a computer-aided drug design process. In light of this, QSAR methodologies, as one of the CADD approaches, are extensively used to accelerate the drug design and discovery process. Successful examples of such drugs are norfloxacin, cimetidine, and zanamivir, originally derived from QSAR analyses [17–20]. In this research, three QSAR-based models were developed and validated. Of these models, one model was derived from the 3D-QSAR method, and the other two were derived from 6D-QSAR analyses. The  conformations needed for 6D-QSAR analyses were obtained from two molecular docking programs, GOLD and AutoDock. By inspecting the performance metrics of the generated models based on training sets, it can be concluded that all of the models have similar results with comparable internal validation statistics (3D-QSAR: R^2^ = 0.98, Q^2^_LOO_ = 0.88; 6D-QSAR (GOLD): R^2^ = 0.92, Q^2^_LOO_ = 0.90; 6D-QSAR (AutoDock): R^2^ = 0.92, Q^2^_LOO_ = 0.89). However, considering the externally validated values, 6D-QSAR models provide significantly better prediction of endpoint values on the unseen data not involved in the training procedure (3D-QSAR: R^2^_Test_ = 0.42; 6D-QSAR (GOLD): R^2^_Test_ = 0.76; (AutoDock): R^2^_Test_ = 0.58). External validation assessment is critical to any QSAR model analysis to propose an efficient model for drug design projects. Considering that the acceptance criterion for the squared correlation coefficient for the test set (i.e., R^2^_Test_) is suggested to be greater than 0.6, the best model for predicting purposes might be the GOLD-based 6D-QSAR model. However, the AutoDock-based 6D-QSAR model can also be marginally acceptable for prediction.

Based on the established rules by Organization for Economic Co-operation and Development (OECD), defining the applicability domain of any QSAR model is inevitable [21]. In this study, AD was determined, and the findings proposed no observation of outliers showing reliability and robustness of the developed 3D- and 6D-QSAR models.

The literature research from 1974 to 2022 includes the QSAR studies with the term “QSAR” in their titles using the Scopus database shows over 9,000 articles. Among these articles, more than 2000 studies have used 3D-QSAR, 75 investigations employed 4D-QSAR, and four and one out of the whole studies were carried out based on 5D- and 6D-QSAR modeling, respectively. However, the remaining ones did not have any dimension value in their titles, suggesting that they would probably belong to dimensions less than or equal to two.

When experimental approaches have not determined a receptor structure, an elegant method for calculating the free energy of ligand binding to target molecules may be found in multi-dimensional QSAR techniques. In light of this viewpoint, 3D-QSAR has been researched using advanced methodologies such as CoMFA, CoMSIA, CoMSA, CoMMA, and many more. The limitations of the 3D-QSAR model have been a matter of debate in several previously reported publications. Due to the limitations of 3D-QSAR, i.e., the selection of correct bioactive conformation of given molecules, more advanced QSAR methods have been developed (e.g., 4D-, 5D-, and 6D-QSAR) [22].

The performance of 3D- and 4D-QSAR approaches were investigated using steric and electronic parameters on CBG binding steroids in work by Polanski et al. Despite discrepancies observed between developed models due to the utilized descriptive codings, CoMFA and 4D-QSAR seemed to produce comparable results [23].

In this study, Bruton’s tyrosine kinase (Btk) inhibitor dataset was employed to assess the performance of QSAR algorithms, including multivariate image analysis (MIA)-QSAR and 4D-QSAR. Analyses of the results indicated the superior performance of 4D-QSAR over MIA-QSAR, with a significant difference in the bioactivity prediction [24].

Findings demonstrate that using appropriate ligand conformers (4D) combined with an induced fit model (5D) is applicable as multi-dimensional QSAR. Vedani et al. have shown that 5D-QSAR is superior to other approaches in estimating the binding affinities of new compounds [25].

Another study investigated different molecular structures of estrogen receptor ligands to predict biological activities. To this end, different levels of QSAR analyses were used, including 3D to 6D-QSAR. Quasar Java-based program was used to develop a 6D-QSAR model considering different conformers of the given ligands, induced-fit models, and solvation models. Comparing cross-validated and predictive r^2^ values demonstrated the superiority of 6D-QSAR to low-dimensional QSAR techniques [26].

Regarding the importance of prediction of the biological activity of the compounds in the early stages of drug design and discovery process before the synthesis of molecules, developing highly predictive models to fulfill this essence can be regarded as a strong point of this study. In this respect, multi-dimensional QSAR may be helpful since it has been evidenced in many recent papers as an innovative strategy. Although considering stereochemistry in multi-dimensional QSAR is an advantage over classic QSAR (i.e., 2D-QSAR), finding appropriate orientation and conformation of the studied ligands is a challenging issue that requires obtaining the available 3D-solved experimental data (X-ray crystallography or NMR data). However, considering the mentioned information, we still need manual intervention and superimposition of ligands for developing multi-dimensional QSAR models.

## Conclusions

QSAR is a ligand-based approach for predicting the biological activity of compounds. This method plays a significant role in the early stages of the drug design and discovery pipeline. With the emergence of more advanced QSAR techniques in terms of dimensionality, substantial progress has been achieved in improving the predictive capability of QSAR-based models. In this work, we investigated the prediction inhibitory activity of human aldose reductase ligands by applying 3D- and 6D-QSAR methods. The findings showed the better performance of the 6D-QSAR model according to the statistical parameters. Overall, an increase in dimensionality in QSAR studies may lead to the development of more reliable QSAR models than in low-dimensional QSAR approaches. However, since scarce studies are available in the literature, more studies are needed to be conducted to verify the findings of this research.

## Methods

### Protein preparation

In this study, a set of X-ray crystal structures in complex with corresponding AR inhibitors were retrieved from the Protein Data Bank (PDB) database (PDB IDs: 1PWM, 1US0, 1Z89, 2PDG, 4PUU, 4PUW, 4Q7B, 4QR6, 2IKG, 2IKH, 2IKI, 2IKJ). Table 4 shows the molecular structures of AR inhibitors used in this investigation. The available reported biological activities of the studied inhibitors (i.e., K_d_) in the literature were obtained with the same experimental conditions covering a range of three orders of magnitude (6.5 nM–7.5 µM) [27–31].

**Table 4:** Chemical structures of AR inhibitors used for QSAR analyses

Comp	PDB	Structure	Refs.	Comp	PDB	Structure	Refs.
a1	1PWM		[27]	a7	4Q7B		[30]
a2	1US0		[28]	a8	4QR6		[30]
a3	1Z89		[28]	a9	2IKG		[31]
a4	2PDG		[29]	a10	2IKH		[31]
a5	4PUU		[30]	a11	2IKI		[31]
a6	4PUW		[30]	a12	2IKJ		[31]

### Molecular docking studies

The co-crystalized AR inhibitors were extracted from the crystal structures and docked into the binding site of AR. GOLD (version 5.2.2; CCDC Inc., Cambridge, UK) [32, 33] and AutoDock (version 4.2) [34] software running under the LINUX operating system were used for molecular docking procedures. The preparation of proteins was performed using in-built modules and tools by applying the default parameters. The hydrogen atoms were added to the protein structures by removing water molecules for both docking protocols. The binding site for molecular docking was defined based on the centroid of the co-crystallized ligands. Semi-flexible docking of studied ligands into the binding site of AR was performed for each ligand. In the case of a GOLD program, all atoms within a radius of 10 Å from the center of the binding site were selected for the docking process by setting the population size of 100, number of operations of 100,000, and number of islands of 5. For AutoDock, Lamarckian Genetic Algorithm (LGA) was applied by accepting default parameters. The obtained solutions were inspected and selected in terms of RMSD to the corresponding reference molecule.

### Multi-dimensional QSAR studies

#### 3D-QSAR study

The 3D coordinates of co-crystallized ligands in complex with AR enzyme were used for a 3D-QSAR study using the Pentacle program (version 1.06) [35]. At first, various probes (i.e., hydrophobic (DRY), hydrogen bond acceptor, HBA (N1), hydrogen bond donor, HBD (O), and shape (TIP) probes) were considered for calculating interaction energies with different parts of the target molecules for obtaining molecular interaction fields (MIFs). For generating the 3D-QSAR model, the data were divided into training and test sets. In this process, we assigned 75% and 25% of the dataset for training and test sets, respectively. Approximately up to 10^5^ nodes were generated, each representing a value for interaction energy. Then, grid-independent descriptors (GRIND) were achieved by calculating node-node interactions to be used as input parameters for 3D-QSAR analysis. The most relevant descriptors significantly contributed to the biological activities were extracted using the AMANDA algorithm for partial least squares (PLS) analysis.

#### 6D-QSAR study

For the 6D-QSAR study, the docked poses with the lowest RMSD compared to the reference molecule were retained as input of the 4D data set in Quasar software (version 6.1) [26, 36]. The same training and test sets used in the 3D-QSAR analysis were employed for the 6D-QSAR approach. Gasteiger-Huckel charges were calculated for the studied molecules using Bio^X^ software (version 4.6) [37]. For the studied molecules, the entropy values were computed. Then, the corresponding induced fit model was created, incorporating the genetic algorithm for optimizing the generation of a quasi-atomistic receptor surface model based on creating 100 models and 5,000 crossover cycles. The Quasar devoted to 6D-QSAR model generation considers a putative receptor site comprised of a three-dimensional surface encompassing the given molecules at a predefined van der Waals distance and mapping the atomistic features onto the generated surface. In 6D-QSAR, different levels of dimensions were taken into account, including the topology property of the binding site, ensemble of conformers, orientations, protonation states of the molecules as the fourth dimension, induced fit parameters as the fifth dimension, and simultaneous incorporation of solvation models as the sixth dimension of QSAR model.

#### Applicability domain

The reliability of the predictive power of the generated QSAR models was evaluated by determining the applicability domain of the studied compounds. For this purpose, the “Williams plot” was generated based on the leverage method, where the Y axis denoted the standardized residuals, and leverage values were expressed by the X axis [38, 39]. In this way, the behavior of each compound is assessed to detect possible outlier(s) in the defined chemical space. The following equation, known as the “Hat” matrix, was used to calculate leverage values (h_i_) of the compounds in the data set:

$${\text{h}}_{\text{i}} = {\text{X}}_{\text{i}}({{\text{X}}^{\text{T}}{\text{X}})}^{-1}{\text{X}}_{\text{i}}^{\text{T}},$$

where X_i_ shows the descriptor matrix of i and X refers to the descriptor matrix of the training set. X^T^ denotes the transpose matrix of X. The warning leverage value (h*) is calculated using the following formula:

$${\text{h}}^{\text{*}}=\frac{3(\text{p}+1)}{\text{n}},$$

where p is the number of descriptors and n represents the number of training compounds. For any compound to be as an outlier, two criteria are considered: compounds with h_i_ greater than warning leverage value (h*) and standard residual of a compound higher than (±) thrice the standard deviation of mean with normally distributed data.

## Supplementary Information

**Additional file 1**. More details and information on 3D-QSAR and 6D-QSAR performance obtained for biological activities of human AR inhibitors.

## Data Availability

All data generated or analysed during this study are included in this published article and its Additional file.

## Abbreviations

CADD: Computer-aided drug design
QSAR: Quantitative structure-activity relationship
AR: Aldose reductase
FFD: Fractional factorial design
3LVs: Three latent variables
SDEP: Standard deviation of the error of prediction
Btk: Bruton’s tyrosine kinase
